# Supplementary material for: Histamine Control in Raw and Processed Tuna: A Rapid Tool Based on NIR Spectroscopy
Source: Foods. 2021 Apr 18;10(4):885. doi: 10.3390/foods10040885 (PMC8074186; doi:10.3390/foods10040885)
Supplement: Supplementary file 1 [file foods-10-00885-s001.zip › foods-1176393 - supplementary.pdf]

SUPPLEMENTARY MATERIAL

NIR spectroscopy as a rapid tool for histamine control in raw and processed tuna

Sergio Ghidini [1], Luca Maria Chiesa [2], Sara Panseri [2,*], Maria Olga Varrà [1], Adriana Ianieri [1], Davide Pessina [3] and Emanuela Zanardi [1]

[1] Department of Food and Drug, University of Parma, Strada del Taglio 10, 43126 - Parma, Italy; sergio.ghidini@unipr.it; mariaolga.varra@unipr.it; adriana.ianieri@unipr.it; emanuela.zanardi@unipr.it
[2] Department of Health, Animal Science and Food Safety, University of Milan, 6 20133 - Milan, Italy; luca.chiesa@unimi.it
[3] Quality Department, Italian Retail Il Gigante SpA, Milan, Italy pessina@ilgigante.net
* Correspondence: sara.panseri@unimi.it

Table S1. Main quantitative and qualitative (discriminant) applications of NIR spectroscopy to different fish and seafood species.

Purpose	Species	Reference
NIR qualitative methodology		
Fresh vs. frozen	Swordfish (Xiphias *gladius*)	[29]
	Cuttlefish (*Sepia officinalis*)	[30]
	Gadidae family (*Gadus morhua, Melanogrammus aeglefinus*)	[31]
Fresh vs. frozen<br>Production method	Cyprinidae family (*Mylopharyngodon piceus, Ctenopharyngodon idellus, Hypophthalmichthys molitrix, Aristichthys nobilis, Cyprinus carpio, Parabramis pekinensis*)	[32]
	Rainbow trout (*Oncorhynchus mykiss*)	[33]
Production method	Sea bass (*Dicentrarchus labrax*)	[34]
Geographical origin	Anchovies (*Engraulis encrasicolus*)	[35]
Geographical origin	Sea cucumber (*Apostichopus japonicus)*	[36]
NIR quantitative methodology		
TVB-N	Grass carp (*Ctenopharyngodon Idella*)	[37]
TBARS and OFR (fluorescence compounds)	Saithe (*Pollachius virens*) and hoki (*Macruronus novaezelandiae*)	[38]
Pigments concentrations	Atlantic salmon (*Salmo salar*)	[39]
Histamine	Tuna	[40]
Proximate composition	Bivalve molluscs (*Saccostrea glomerata, Ostrea angasi, Crassostrea gigas, Mytilus galloprovincialis, Anadara trapezia)*	[41]
	Tilapia	[42]
Fatty acids classes	Atlantic salmon (*Salmo salar*)	[43]
Sodium chloride concentration	Atlantic salmon (*Salmo salar*)	[44]
TMA-N	Silver carp (*Hypophthalmichthys molitrix*)	[45]
Texture profile analysis (TPA) parameters	Crucian carp (*Carassius carassius*)	[46]
Drip loss and pH	Atlantic salmon (*Salmo salar)*	[47]
Freshness (days)	Atlantic salmon (*Salmo salar*)	[48]
Color attributes (L*, a*, b*)	Atlantic salmon (*Salmo salar*)	[49]
Microbial loads	Rainbow trout (*Oncorhynchus mykiss*)	[50]
Cadaverine	Chub mackerel (*Scomber japonicus)*	[51]
